# Improved pseudocapacitive charge storage in highly ordered mesoporous TiO_2_/carbon nanocomposites as high-performance Li-ion hybrid supercapacitor anodes†

**DOI:** 10.1039/c9ra07157a

**Published:** 2019-11-20

**Authors:** Yujin Lee, Seoa Kim, Jeong Han Lee, Kwang Chul Roh, Eunho Lim, Jinwoo Lee

**Affiliations:** Department of Chemical and Biomolecular Engineering, Korea Advanced Institute of Science Technology (KAIST) Daejeon 34141 Republic of Korea jwlee1@kaist.ac.kr; Energy and Environmental Division, Korea Institute of Ceramic Engineering and Technology (KICET) Jinju Gyeongnam 52851 Republic of Korea; Carbon Resources Institute, Korea Research Institute of Chemical Technology (KRICT) Daejeon 34114 Republic of Korea eunholim@krict.re.kr

## Abstract

A Li-ion hybrid supercapacitor (Li-HSCs), an integrated system of a Li-ion battery and a supercapacitor, is an important energy-storage device because of its outstanding energy and power as well as long-term cycle life. In this work, we propose an attractive material (a mesoporous anatase titanium dioxide/carbon hybrid material, m-TiO_2_-C) as a rapid and stable Li^+^ storage anode material for Li-HSCs. m-TiO_2_-C exhibits high specific capacity (∼198 mA h g^−1^ at 0.05 A g^−1^) and promising rate performance (∼90 mA h g^−1^ at 5 A g^−1^) with stable cyclability, resulting from the well-designed porous structure with nanocrystalline anatase TiO_2_ and conductive carbon. Thereby, it is demonstrated that a Li-HSC system using a m-TiO_2_-C anode provides high energy and power (∼63 W h kg^−1^, and ∼4044 W kg^−1^).

## Abstract 2

A mesoporous TiO_2_/carbon nanocomposite prepared by block copolymer self-assembly improves pseudocapacitive behavior and achieves high energy/power density Li-ion hybrid supercapacitors.

## Graphical abstract

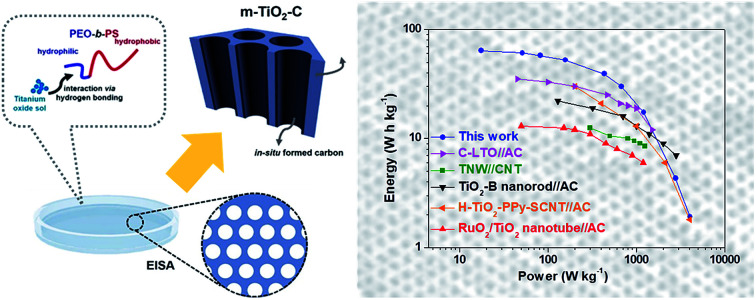

## Introduction

High-power energy-storage devices have been regarded as indispensable systems for medium- and large-scale energy-storage applications such as electric vehicles (EVs) and smart grid technologies. This is because the medium- and large-scale energy storage applications require fast charging and discharging behaviors with long cycle lifetime.^1,2^ An electric double-layer capacitor (EDLC), which is one of the typical supercapacitors, is the representative energy-storage device that provides highly excellent power abilities (2–5 kW kg^−1^) with stable cyclability.^3–6^ However, in spite of its superb abilities, it suffers from low energy performance (∼10 W h kg^−1^) due to its charge-storage mechanism being based on the physisorption of solvated ions at electrode/electrolyte interfaces.^7–9^

As its alternative device, Li-ion hybrid supercapacitors (Li-HSCs) composed of Li-ion battery (LIB) electrode materials as anodes and EDLC electrode materials as cathodes in a non-aqueous electrolyte containing a Li salt have been intensively researched and developed in recent years.^10–12^ This is because that their electrochemical performance delivering both high energy and power abilities with stable cyclability is highly attractive. Such unique characteristics result from asymmetric charge-storage mechanisms that Li^+^ from the electrolyte inserts to the anode materials (faradaic reaction) and anions such as PF_6_^−^ and ClO_4_^−^ are physically adsorbed on the surface of the cathode materials (non-faradaic reaction) during the charge process.^10^ However, because of the different charge-storage mechanisms between two electrodes, a kinetics imbalance issue is one of the major problems in Li-HSCs, which is should be solved to develop high-performance Li-HSCs.^13,14^ In other words, the reaction mechanism of the anode materials using ionic diffusion in a crystal framework of electrode materials is much more sluggish than that of cathode materials.^15,16^

To address this kinetics issue, two kinds of strategies have intensively been considered. One of the effective strategies is to reduce particle size of electrode materials in the nanoscale regime.^15^ Well-nanosized electrode materials have a variety of merits such as shortened lengths for Li^+^ diffusion/electron mobility (high-power ability) and enhanced electrode/electrolyte interface area (high capacity).^17–19^ In addition, reducing particle size of electrode materials in the nanoscale leads to improved pseudocapacitive reactions associated with surface-controlled reactions, which is kinetically not limited by diffusion-controlled reactions.^20^ Another strategy is to use attractive carbonaceous and Ti-based anode materials such as graphite and titanium dioxide (TiO_2_).^21–24^ Particularly, anatase TiO_2_ is one of the promising anode materials for Li-HSCs, because of its beneficial Li^+^ insertion/extraction behaviors and a variety of merits. Firstly, it provides theoretical capacity of ∼168 mA h g^−1^ in the potential range of ∼1.7 V (*vs.* Li/Li^+^) with small volume change (<4%) during cycling.^21,25^ Therefore, compared to the graphite anode working in the potential range of ∼0.2 V (*vs.* Li/Li^+^), it is free from Li-plating problem and can provide highly stable cycle performance.^26,27^ Secondly, Li-HSCs using anatase TiO_2_ anodes can use a cost-attractive and lightweight Al current collector in the anodic part instead of Cu current collector because alloying reaction between Li^+^ and Al does not take place in the potential range of 1.0–3.0 V (*vs.* Li/Li^+^) for anatase TiO_2_. In addition, it is non-toxic and one of the highly abundant materials.^28,29^ However, the drawback of anatase TiO_2_ is its poor electrical conductivity and relatively low Li^+^ diffusion rate.^28,30,31^

Here, to rationally design the anatase TiO_2_ as anode materials for Li-HSCs, we synthesized mesoporous anatase TiO_2_/carbon nanocomposite (denote as m-TiO_2_-C) by using block copolymer assisted simple synthesis method. It is clearly demonstrated in this work that design of the synthesized mesoporous electrode material comprising both nanocrystalline anatase TiO_2_ and conductive carbon is highly appropriate to solve drawback of the anatase TiO_2_ and to maximize its electrochemical performance (high capacity and rate capability), which result from improved Li^+^ diffusion kinetics with rapid electron transport. Furthermore, we proved that the well-designed m-TiO_2_-C is highly attractive as anode materials for Li-HSCs delivering high energy and power.

## Experimental

### Synthesis of m-TiO_2_-C

0.15 g of PEO-*b*-PS (poly(ethylene oxide)-*b*-poly(styrene), *M*_n_ = 27 466 g mol^−1^) was dissolved in 4 mL tetrahydrofuran (THF). 0.9 mL of titanium isopropoxide (TTIP) with 0.3 mL of 35–37% hydrochloric acid (concentrated HCl) was added dropwise to the block copolymer/THF mixture solution with continuous stirring. After 1 h of stirring, the mixture solution was poured to the glass Petri dish. The solvent was evaporated overnight at 40 °C, and then dried at 100 °C. The as-synthesized transparent film was obtained and then, the collected power was heat-treated in Ar atmosphere at 700 °C for 2 h. Commercial TiO_2_ purchased from Sigma Aldrich was employed for control group (denoted as com-TiO_2_).

### Materials characterization

#### Structure and chemical characterization

The structure and morphology of prepared samples were investigated using scanning electron microscopy (SEM; S-4200 field-emission, Hitachi) and high resolution-transmission electron microscopy (HR-TEM; JEOL JEM-2010). Nitrogen adsorption–desorption analysis was conducted with 77 K with Micromeritics Tristar II 3020 system to estimate pore size and specific surface area. To confirm the specific pore morphology, small-angle X-ray scattering (SAXS) patterns was detected using 4C SAXS beamlines at the Pohang Light Source (PLS). To investigate crystalline phase, X-ray diffraction (XRD) pattern was identified by D/max-2500 a diffractometer (Rigaku, Cu-Kα radiation). The carbon content of m-TiO_2_-C was estimated using thermogravimetric analysis (TGA; NETZSCH STA 449C). Electron energy loss spectroscopy (EELS) analysis was performed to identify containing elements using energy-filtering transmission electron microscopy (EF-TEM, JM-220FS).

#### Electrochemical measurements

For half and full-cell electrochemical tests, active materials including m-TiO_2_-C and com-TiO_2_ (80 wt%) were homogeneously mixed with super-P carbon (10 wt%) and polyvinyledene fluoride (PVDF, 10 wt%) in *N*-methyl-2-pyrrolidone (NMP). The slurries were pasted on current collector using doctor blade. The prepared electrodes were dried at 60 °C for 6 h and then, 110 °C for 12 h in vacuum oven. Subsequently, the electrodes were roll-pressed. For half-cell test, 2032-type coin cells were fabricated with lithium metal as both counter and reference electrodes in Ar-filled glovebox. The mass loading of active materials used as anode materials were carefully controlled around 1.0 mg cm^−2^. The electrolyte was 1.0 M of LiPF_6_ in mixture of ethylene carbonate/dimethyl carbonate (EC/DMC, 1 : 1 volume ratio, Panaxetec. Co., Korea). The activated carbon electrode used as cathode materials was prepared using commercially available MSP-20 (90 wt%), conductive carbon (5 wt%), and polytetrafluoroethylene (PTFE, 5 wt%). In the half-cell tests, the working voltages for anode and cathode materials were 1.0–3.0 and 3.0–4.5 V (*vs.* Li/Li^+^), respectively. In the case of full-cell, Li-HSC was assembled using m-TiO_2_-C as an anode and MSP-20 as a cathode and the weight ratio of anode and cathode active materials was controlled to 1 : 3.5 in working voltages of 0–3.0 V. The energy and power of the Li-HSCs was calculated by numerically integrating the galvanostatic discharge profiles using eqn (1) and (2) as follows.^32^1
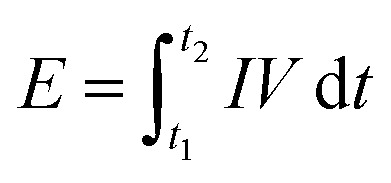
where *I* is the constant current (A g^−1^), *V* is the working voltage (V), *t*_1_ and *t*_2_ are the start/end of discharge time (s) of Li-HSCs, respectively, and2
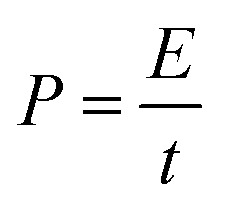
where *t* is the discharge time (s). All electrochemical tests were conducted using the WBCS-3000 battery cycler (WonA Tech, Korea).

## Results and discussion

### Material characterization

**Fig. 1:**
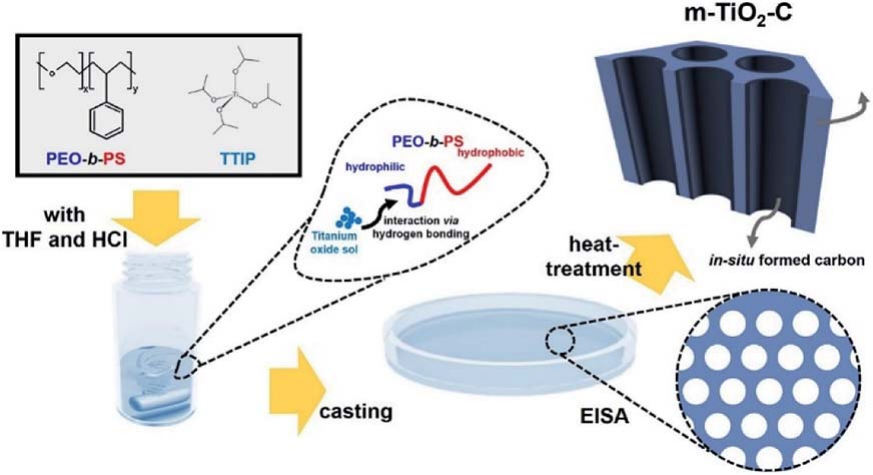
Schematic illustration of the synthesis of m-TiO_2_-C.

The m-TiO_2_-C was synthesized using a block copolymer-assisted synthesis method and schematic representation on the synthesis method is shown in Fig. 1. In brief, the Ti precursor (TTIP) and laboratory-made amphiphilic block copolymer (poly(ethylene oxide)-*b*-poly(styrene), PEO-*b*-PS) synthesized by atomic transfer radical polymerization (ATRP) were dissolved in tetrahydrofuran (THF). And then, to induce sol–gel reaction of TTIP, concentrated hydrochloric acid (HCl) was slowly added to the TTIP/block copolymer/THF mixture solution. The titanium oxide sol made by hydrolysis selectively interacts with the hydrophilic PEO part of PEO-*b*-PS *via* hydrogen bonds. During the evaporation of organic solvent at 40 °C (evaporation-induced self-assembly, ESIA), highly ordered mesostructure is formed by self-organization of titanium oxide sol/block copolymer mixture.^33–36^ After drying at 100 °C to induce the cross-linkage of titanium oxide sol, the as-synthesized TiO_2_/block copolymer composite was heat-treated at 700 °C under inert atmosphere (Ar condition). During heat-treatment, as-synthesized TiO_2_ is converted to crystalline TiO_2_. In addition, PS part of PEO-*b*-PS is converted to mechanically stable and conductive carbon (*in situ* formed carbon in m-TiO_2_-C). For comparison, commercially available TiO_2_ (com-TiO_2_) was employed.

**Fig. 2:**
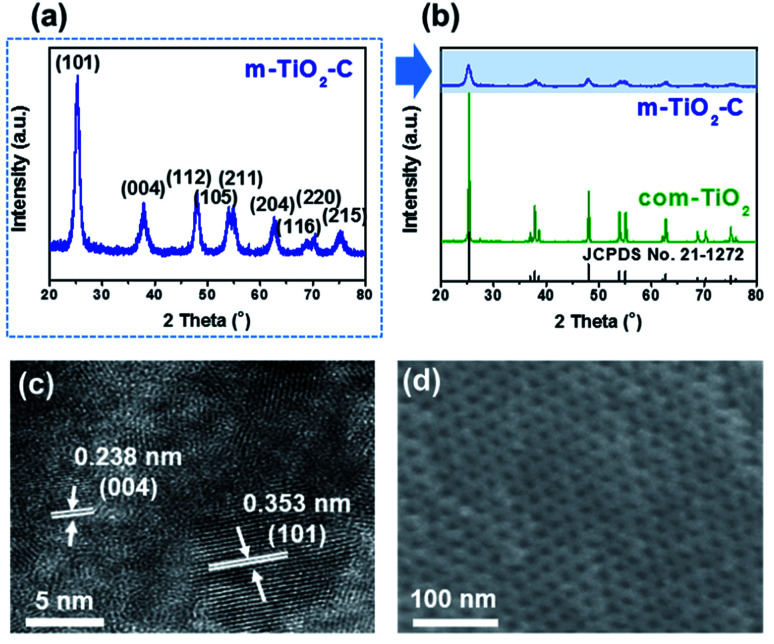
(a) XRD pattern on m-TiO_2_-C. (b) Comparison of XRD patterns on m-TiO_2_-C and com-TiO_2_. (c) HR-TEM image of m-TiO_2_-C. (d) SEM image of m-TiO_2_-C.

**Fig. 3:**
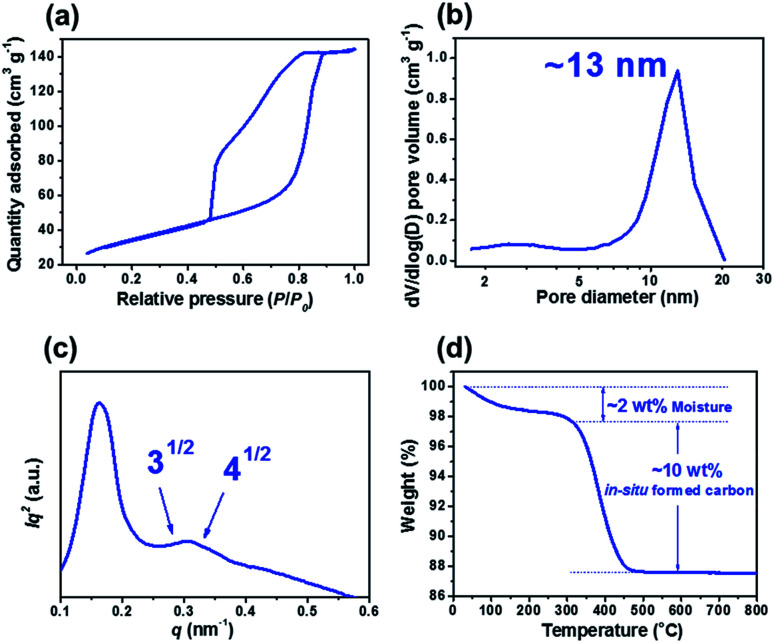
(a) N_2_ physisorption isotherm and (b) pore size distribution of m-TiO_2_-C. (c) SAXS pattern of m-TiO_2_-C. (d) TGA result of m-TiO_2_-C.

**Fig. 4:**
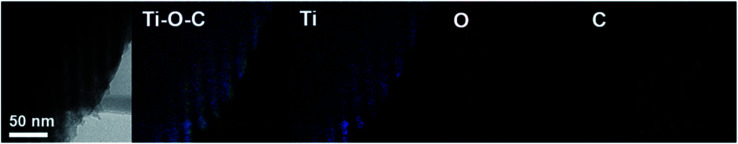
EELS mapping images of m-TiO_2_-C.

Fig. 2a and b show that X-ray diffraction (XRD) patterns of m-TiO_2_-C and com-TiO_2_ are in good agreement with anatase TiO_2_ (JCPDS no. 21-1272) with no noticeable impurities. Their average crystallite sizes calculated using the Debye–Scherrer equation were ∼14 (m-TiO_2_-C) and ∼100 (com-TiO_2_) nm, respectively.^37^ In addition, high-resolution transmission electron microscopy (HR-TEM, Fig. 2c) image of m-TiO_2_-C clearly shows the lattice fringes of anatase TiO_2_ with (101) and (004) spacing of 0.353 and 0.238 nm, respectively, again indicating that the crystal structure of m-TiO_2_-C well match the anatase TiO_2_ phase. Mesoporous structure of m-TiO_2_-C is identified through scanning electron microscopy (SEM) image, N_2_ adsorption–desorption technique, and small-angle X-ray scattering (SAXS) patterns. SEM image (Fig. 2d) shows highly ordered mesoporous structure with uniform pore size. Compared to m-TiO_2_-C, com-TiO_2_ has irregular and larger particle shape/size without porosity (Fig. S1†). In addition, N_2_ adsorption isotherm (Fig. 3a) of m-TiO_2_-C corresponds to type IV curve with a sharp adsorption at ∼0.9 *P*/*P*_0_, indicating that uniform mesopores are predominant. The main pore size calculated using the Barret–Joyner–Halenda (BJH) method and specific surface area calculated using the Brunauer–Emmett–Teller (BET) of m-TiO_2_-C were ∼13 nm and ∼123 m^2^ g^−1^ (Fig. 3b), respectively, which is significantly higher than that of com-TiO_2_ (<2 m^2^ g^−1^). The mesoporous structural characterization of m-TiO_2_-C is further demonstrated by SAXS pattern (Fig. 3c). Scattering peaks of m-TiO_2_-C with a peak position ratio of 1 : 3^1/2^ : 4^1/2^ suggest that hexagonally ordered TiO_2_ structure with a long-range order is well established.^38^ To confirm the presence of *in situ* formed carbon in m-TiO_2_-C, thermogravimetric analysis (TGA) and electron energy loss spectroscopy (EELS) analysis were employed. TGA result (Fig. 3d) proves that the *in situ* formed carbon content in the m-TiO_2_-C is around 10 wt%. Furthermore, EELS analysis image (Fig. 4) directly shows the existence of *in situ* formed carbon in the m-TiO_2_-C, representing that Ti, O, and C are uniformly dispersed.

### Electrochemistry

**Fig. 5:**
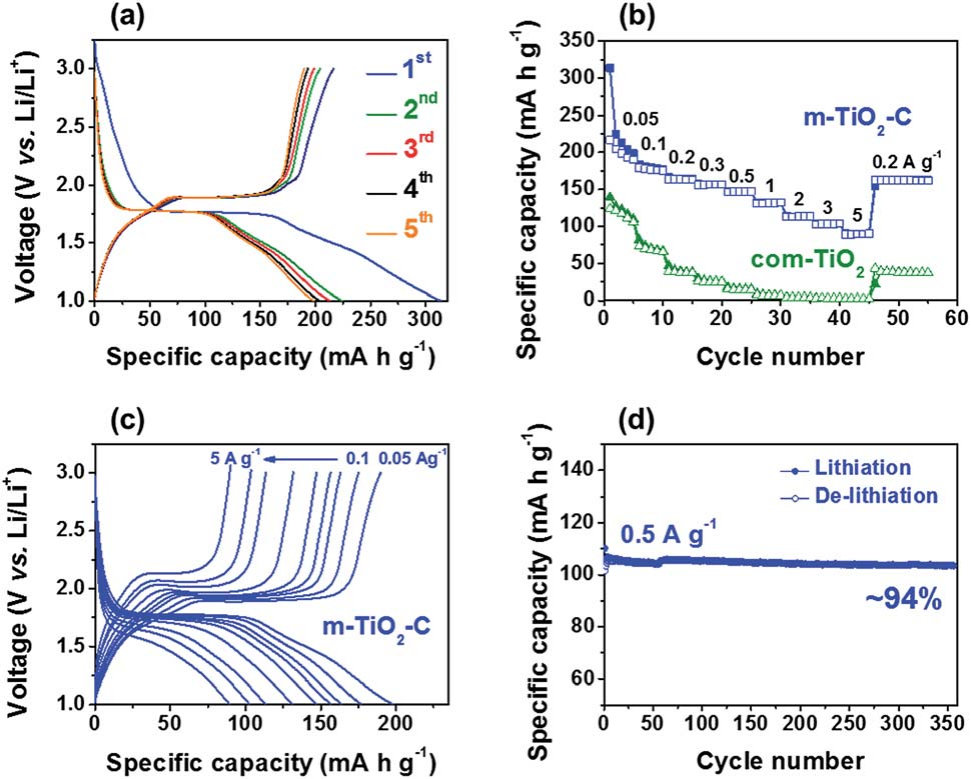
(a) Galvanostatic charge–discharge profiles of m-TiO_2_-C at 0.05 A g^−1^. (b) Comparison of rate capability of TiO_2_ electrodes at different currents from 0.05 to 5 A g^−1^. (c) Galvanostatic charge–discharge profiles of m-TiO_2_-C at various currents from 0.05 to 5 A g^−1^. (d) Cycle performance of m-TiO_2_-C at a current of 0.5 A g^−1^.

Galvanostatic charge–discharge (de-lithiation and lithiation) test on m-TiO_2_-C was conducted in the potential range of 1.0–3.0 V (*vs.* Li/Li^+^), showing that the m-TiO_2_-C provides reversible charge capacity of ∼198 mA h g^−1^ at current of 0.05 A g^−1^ (Fig. 5a). The typical plateau at a potential of ∼1.7 V (*vs.* Li/Li^+^) demonstrates reversible Li^+^ intercalation into anatase TiO_2_ lattice, where TiO_2_ + *x*Li^+^ + *x*e^−^ ↔ Li_*x*_TiO_2_ (0 ≤ *x* ≤ 0.5).^22,28,39^Fig. 5b shows the specific capacity of m-TiO_2_-C is much higher (∼198 mA h g^−1^) than that of com-TiO_2_ (∼110 mA h g^−1^) at current of 0.05 A g^−1^. In addition, compared to com-TiO_2_, the m-TiO_2_-C showed much better rate capability with increasing currents from 0.05 to 5 A g^−1^, also indicating that capacity retention of m-TiO_2_-C with increasing currents is significantly outstanding than that of com-TiO_2_ (Fig. S2a†). Fig. 5c and S2b† shows that the galvanostatic charge–discharge curves of m-TiO_2_-C with increasing currents are well maintained with small overpotentials (reduced internal resistance) compared to those of com-TiO_2_. It represents that well-ordered structure with conductive *in situ* formed carbon is highly beneficial to improve electrochemical performances of anatase TiO_2_, mainly due to a variety of merits including (i) shortened diffusion lengths of Li^+^, (ii) superior electron mobility, (iii) easy penetration of electrolyte, (iv) plentiful charge-storage sites, and *etc.*^34,40–42^ It should be noted that the m-TiO_2_-C exhibits better rate capability than other anatase TiO_2_ anodes previously reported (Fig. S3†) and delivers highly stable cycle stability (capacity retention of ∼94% with ∼100% coulombic efficiency for ∼350 cycles) at the current of 0.5 A g^−1^ (Fig. 5d and S4†).^43–47^ In addition, it shows stable long-term cyclability at high current of 3 A g^−1^ for 1000 cycles (Fig. S4,† capacity retention of ∼97% with ∼100% coulombic efficiency). Because the excellent rate capability and long-term cycle stability are main factors for application to anodes of Li-HSCs, m-TiO_2_-C developed in this work could be the extremely potential Li-HSC anode material.

**Fig. 6:**
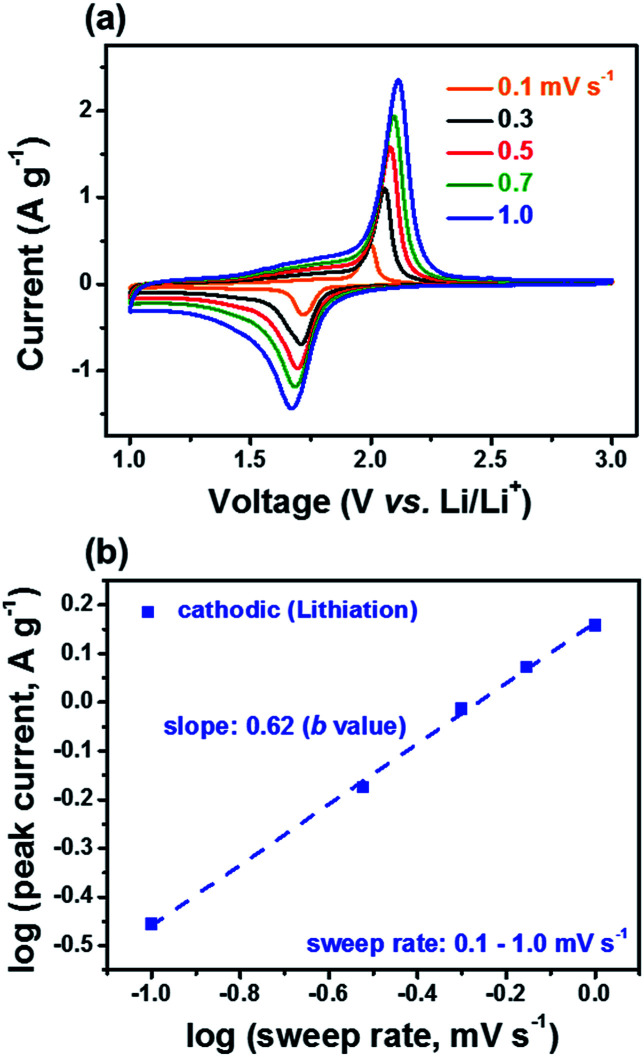
(a) CV curves of m-TiO_2_-C at different sweep rates of 0.1–1.0 mV s^−1^. (b) log(*i*) *vs.* log(*v*) plot of cathodic peak current on m-TiO_2_-C electrode.

To further reveal the reason why m-TiO_2_-C provides superior electrochemical behaviors, we performed cyclic voltammetry (CV) tests on m-TiO_2_-C and com-TiO_2_ conducted at sweep rates from 0.1 to 1.0 mV s^−1^ in the potential range of 1.0–3.0 V (*vs.* Li/Li^+^) as shown in Fig. 6a and S5.† The pair of redox peaks (1.7–2.0 V *vs.* Li/Li^+^) of m-TiO_2_-C and com-TiO_2_ at sweep rate of 0.1 mV s^−1^ well match the lithiation and de-lithiation reactions. In previous studies, it is well known that the anatase TiO_2_ is influenced by the diffusion-controlled process with a two-phase process. Therefore, from *i* = *av*^*b*^ equation (where *a* (mV s^−1^)) with CV tests, *b* value of anatase TiO_2_ is close to 0.5, indicating that the diffusion-controlled reactions is more dominant than the surface-controlled reaction (*b* = 1.0).^20,48,49^ As shown in Fig. 6b and S6,†*b* value of m-TiO_2_-C obtained from peak currents of the lithiation process is around 0.62, indicative of that the charge-storage reaction mechanism in m-TiO_2_-C is influenced by both the surface- and diffusion-controlled reactions (improved pseudocapacitive reaction).^49^ It is considerably contrast to that of com-TiO_2_ showing severe deviation with increasing sweep rates from 0.1 to 1.0 mV s^−1^ because of huge internal resistance. In addition, *b* values of m-TiO_2_-C obtained in potential range of 1.2–2.1 V (*vs.* Li/Li^+^) are also higher than 0.5 (Fig. S6†).

**Fig. 7:**
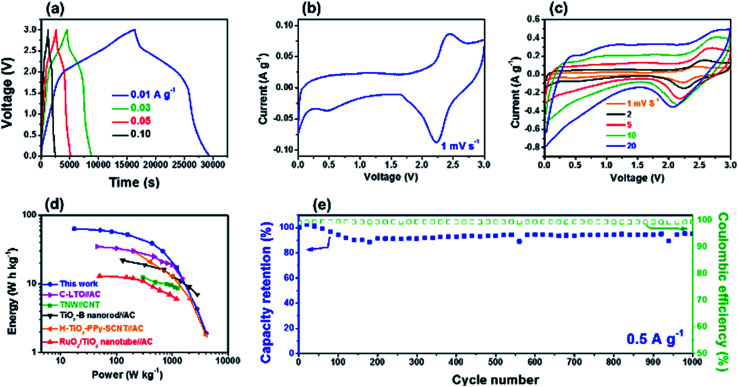
(a) Galvanostatic charge–discharge profiles of the Li-HSC at different currents from 0.01 to 0.1 A g^−1^. (b and c) CV curves of the Li-HSCs at different sweep rates of 1–20 mV s^−1^. (d) Ragone plots compared with previously reported results. (e) Cycle performance of the Li-HSC at a current of 0.5 A g^−1^.

Before building Li-HSC system using the m-TiO_2_-C anode, electrochemical behavior of MSP-20 (commercially available activated carbon) as a cathode material for the Li-HSC was investigated by galvanostatic charge–discharge half-cell test. Fig. S7† shows that reversible specific capacity of the MSP-20 is ∼60 mA h g^−1^ at a current of 0.05 A g^−1^ in the voltage range of 3.0–4.5 V (*vs.* Li/Li^+^). Considering the specific capacities and working voltages between m-TiO_2_-C anode and MSP-20 cathode, the Li-HSC system using m-TiO_2_-C anode and MSP-20 cathode was carefully assembled and its galvanostatic charge–discharge tests were conducted at various currents in the potential range of 0.0–3.0 V (Fig. 7a and S8†). Galvanostatic charge–discharge curves of Li-HSC using m-TiO_2_-C as anode and MSP-20 as cathode at current rates from 0.01 to 5 A g^−1^ do not exhibit typical triangular shape, dissimilar to those of conventional symmetric SCs.^50^ It is again confirmed from CV data at sweep rates of 1–20 mV s^−1^ (Fig. 7b and c) that the CV profile does not follows a typical rectangular shape of conventional symmetric SCs. The galvanostatic charge–discharge and CV shapes of the Li-HSC are mainly due to combination of the faradaic reaction at the m-TiO_2_-C anode and the non-faradaic reaction at the MSP-20 cathode.^22^ The maximum energy and power of the Li-HSC calculated using eqn (1) and (2) with galvanostatic charge–discharge curves were ∼63 W h kg^−1^ and ∼4044 W kg^−1^, respectively. The Ragone plot on the trade of relationship between energy and combination of the faradaic reaction at the m-TiO_2_-C anode and the non-faradaic reaction at the MSP-20 cathode.^22^ The maximum energy and power of the Li-HSC calculated using eqn (1) and (2) with galvanostatic charge–discharge curves were ∼63 W h kg^−1^ and ∼4044 W kg^−1^, respectively. The Ragone plot on the trade of relationship between energy and power shows that its energy and power is much better than that of other results previously reported (Fig. 7d).^43,51–54^ Finally, long-term cycle stability of the Li-HSC was investigated. Fig. 7e shows that the cycling stability of the Li-HSC at a current of 0.5 A g^−1^ is well maintained with ∼100% coulombic efficiency for 1000 cycles. The Ragone plot and cycle performance data imply that m-TiO_2_-C is highly suitable as the anode material for Li-HSC system.

## Conclusions

In summary, we reported the block copolymer assisted one-pot synthesis method of m-TiO_2_-C and its application for high-power anode materials of Li-HSC. The m-TiO_2_-C delivered high specific capacity (∼198 mA h g^−1^ at 0.05 A g^−1^) and rate capability (∼90 mA h g^−1^ at 5 A g^−1^) with stable cycle performance. Its electrochemical performance was superior compared to com-TiO_2_, mainly due to synergistic effects of unique mesostructure and hybridization between anatase TiO_2_ and *in situ* formed carbon. Thereby, the Li-HSC system using the m-TiO_2_-C anode possessed high energy and power abilities (∼63 W h kg^−1^ and ∼4044 W kg^−1^) in the potential range of 0.0–3.0 V, implying that the energy-storage device (Li-HSC) can be a promising alternative to conventional symmetric SCs.

## Conflicts of interest

There are no conflicts to declare.

## Supplementary Material

RA-009-C9RA07157A-s001
