# Supplementary material for: Improved pseudocapacitive charge storage in highly ordered mesoporous TiO2/carbon nanocomposites as high-performance Li-ion hybrid supercapacitor anodes
Source: RSC Adv. 2019 Nov 20;9(65):37882–8. doi: 10.1039/c9ra07157a (PMC9075818; doi:10.1039/c9ra07157a)
Supplement: RA-009-C9RA07157A-s001 [file RA-009-C9RA07157A-s001.pdf]

Electronic Supplementary Material (ESI) for RSC Advances.
This journal is © The Royal Society of Chemistry 2019

Supporting Information

Improved Pseudocapacitive Charge Storage in Highly Ordered Mesoporous TiO_2/Carbon Nanocomposites as High-Performance Li-Ion Hybrid Supercapacitor Anodes

Yujin Lee,[a] Seoa Kim,[a] Jeong Han Lee,[b] Kwang Chul Roh,[b] Eunho Lim,*[c] and Jinwoo Lee*[a]

[a]Department of Chemical and Biomolecular Engineering, Korea Advanced Institute of Science Technology (KAIST), Daejeon 34141, Republic of Korea

[b]Energy and Environmental Division, Korea Institute of Ceramic Engineering and Technology (KICET), Jinju, Gyeongnam 52851, Republic of Korea

[c]Carbon Resources Institute, Korea Research Institute of Chemical Technology (KRICT), Daejeon 34114, Republic of Korea

E-mail: eunholim@krict.re.kr and jwlee1@kaist.ac.kr

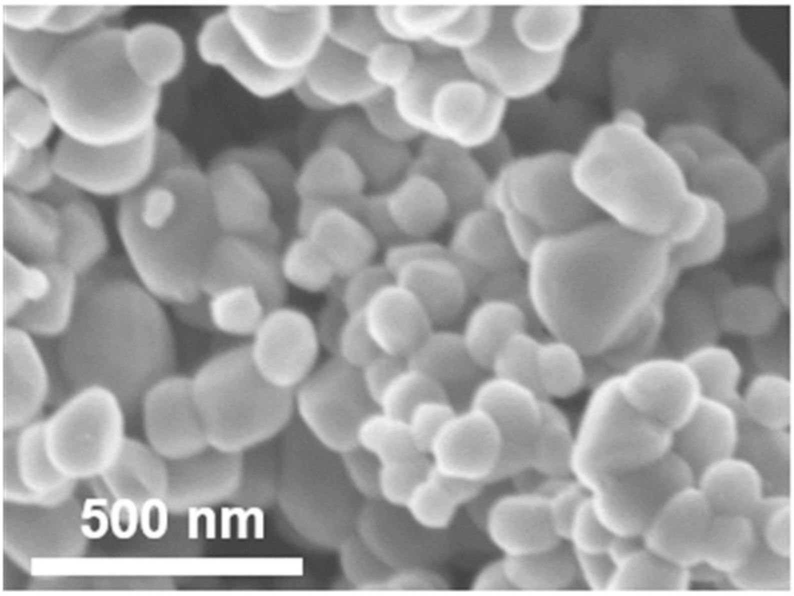

Figure S1. SEM image of com-TiO_2.

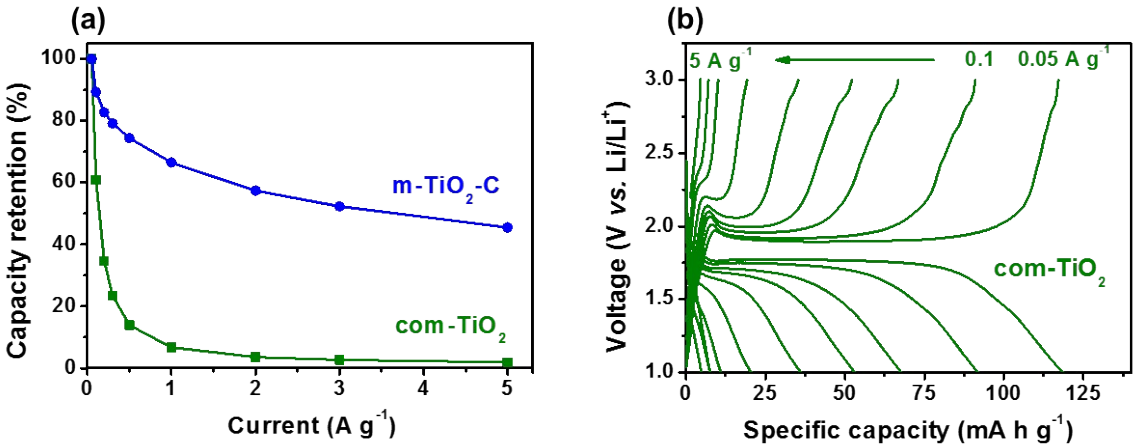

Figure S2. (a) Comparison of capacity retention of TiO_2 electrodes at different currents from 0.05 to 5 A g^{-1}. (c) Galvanostatic charge-discharge profiles of com-TiO_2 at various currents from 0.05 to 5 A g^{-1}.

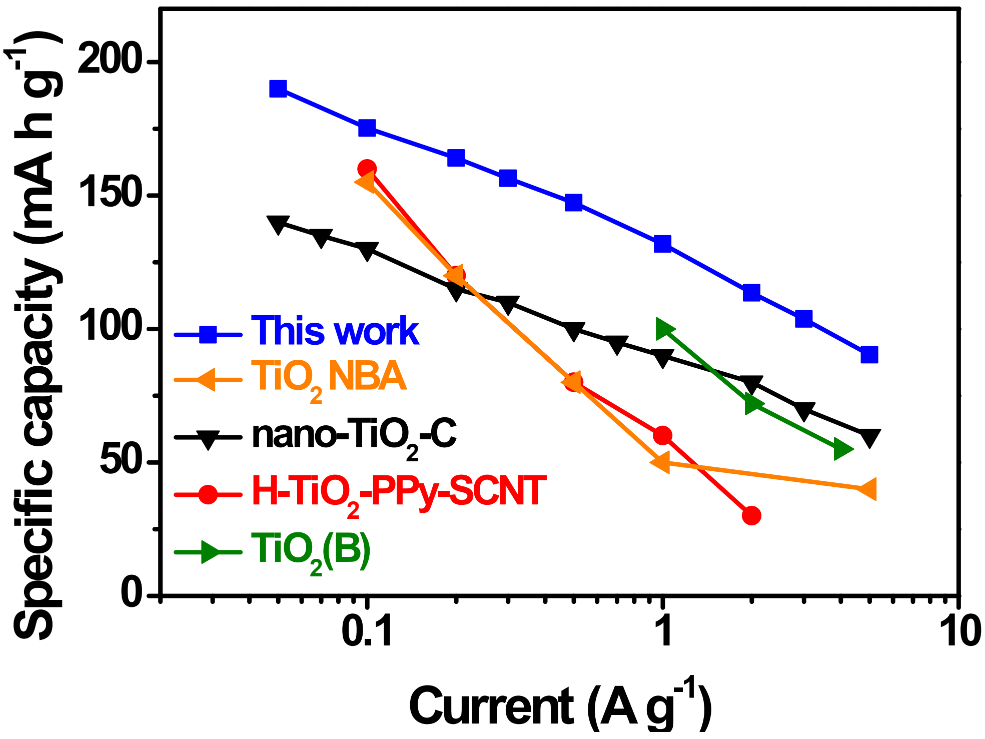

Figure S3. Comparison of rate capability on m-TiO_2-C and previously reported results.

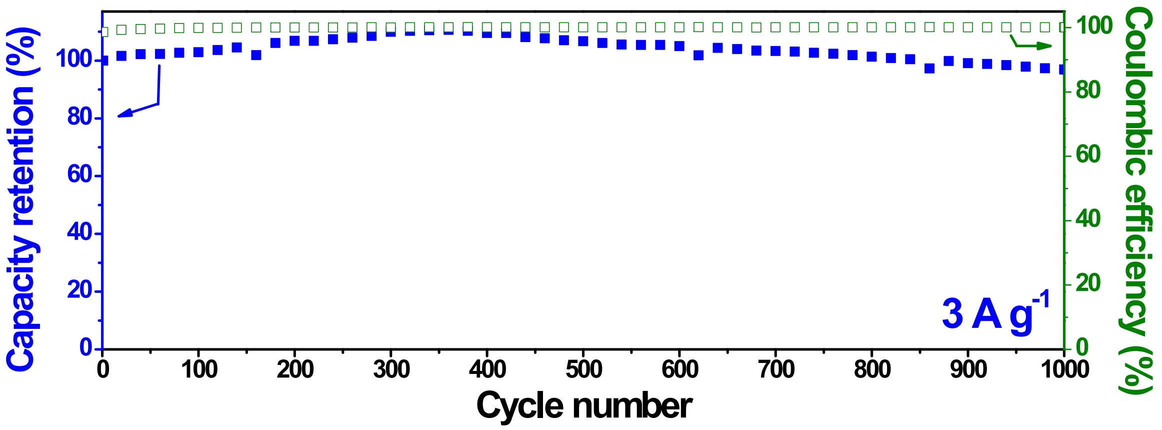

Figure S4. Cycle performance of m-TiO_2-C at a current of 3 A g^{-1}.

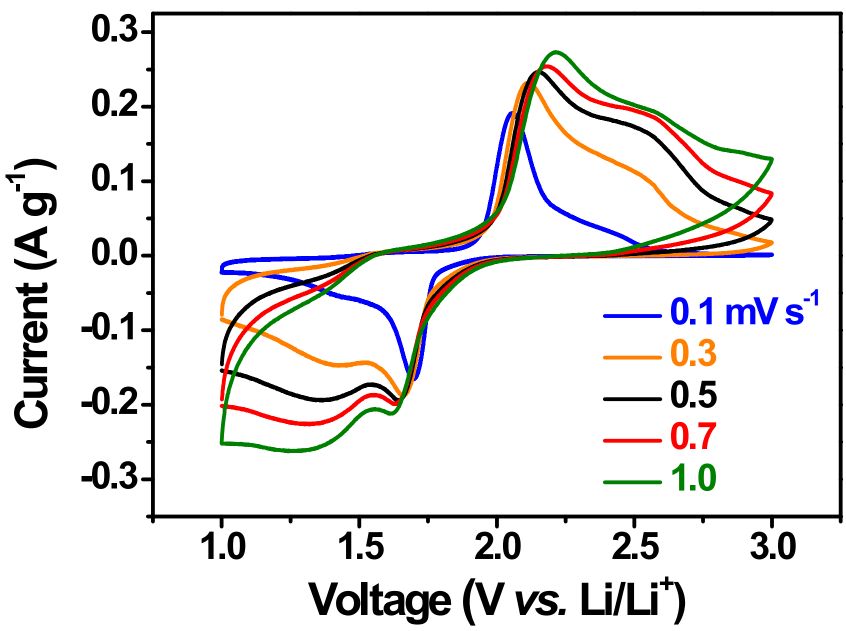

Figure S5. CV curves of com-TiO_2 at different sweep rates of 0.1-1.0 mV s^{-1}.

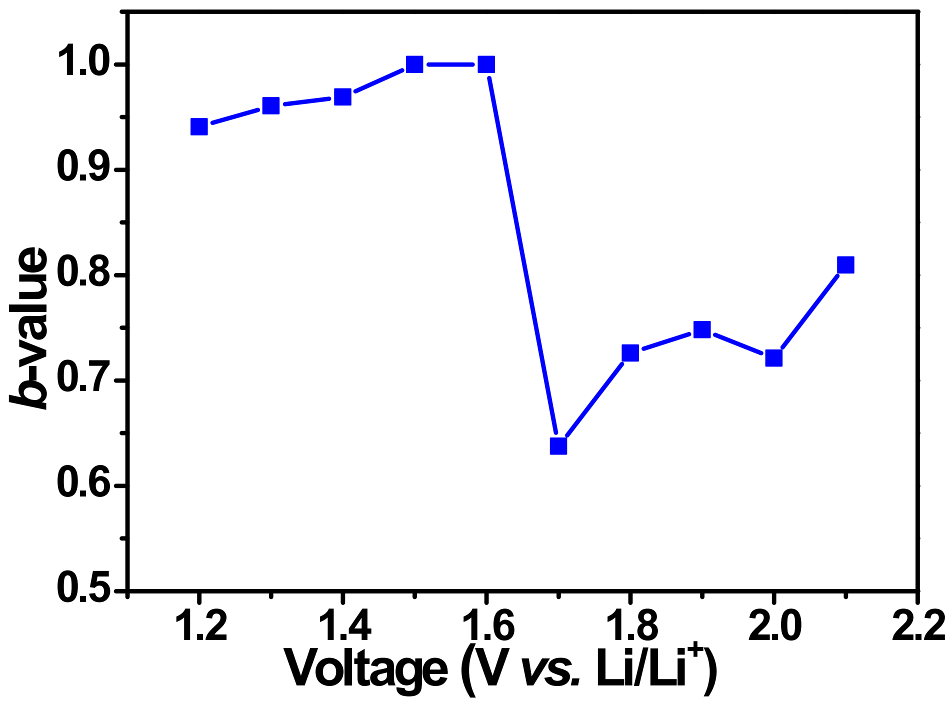

Figure S6. *b* values of m-TiO_2-C plotted as a function of potential for cathodic sweeps.

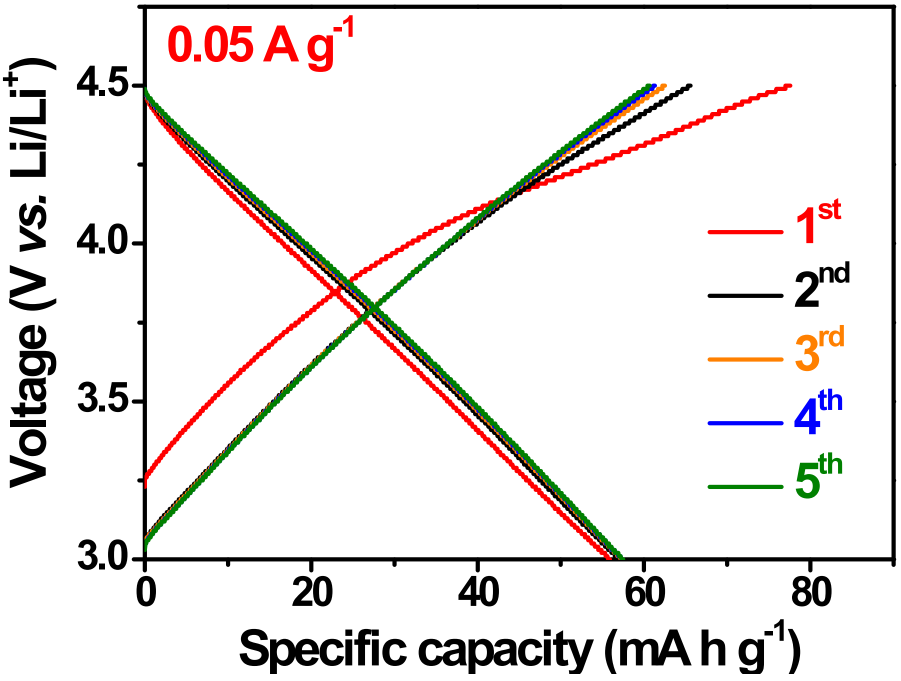

Figure S7. Galvanostatic charge-discharge profiles of MSP-20 at 0.05 A g^{-1}.

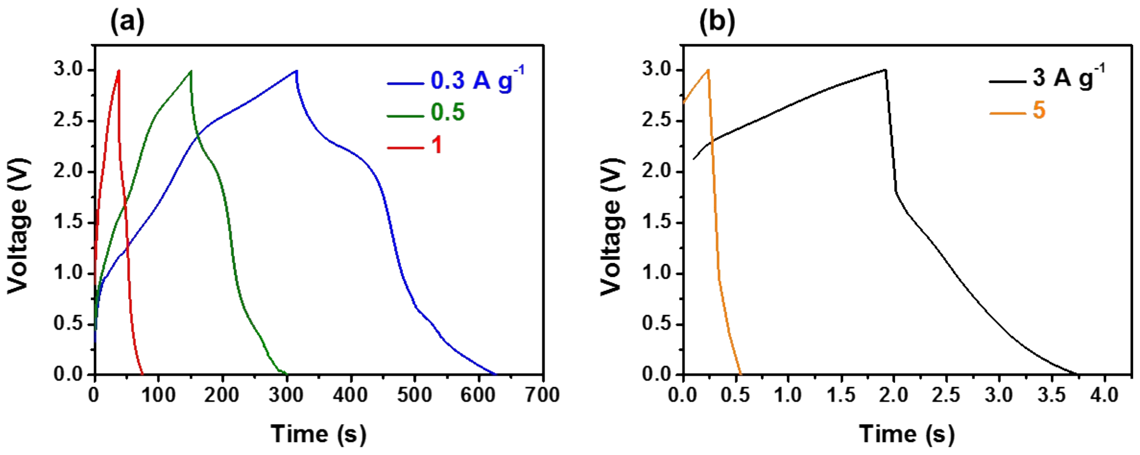

Figure S8. Galvanostatic charge-discharge profiles of the Li-HSC at different currents from (a) 0.3 to 1 A g^{-1} and from (b) 3 to 5 A g^{-1}.

Table S1. Comparison of electrochemical performances of Li-HSC using m-TiO_2-C (this work) with other Li-HSC results previously reported.

Sample		Energy (W h kg^{-1})	Power (W kg^{-1})	Voltage range (V)	Electrolyte	Ref.
**Anode**	**Cathode**					
**m-TiO_2-C**	**AC**	~63	~4,044	0 – 3.0	1.0 M $LiPF_6$ in EC/DMC	This work
**C-LTO**	**AC**	~36	~1,500	1.5 – 2.5	1.0 M $LiPF_6$ in PC	[51]
**TNW**	**CNT**	~13	~1,300	0 – 2.8	1.0 M $LiPF_6$ in EC/DMC	[52]
**TiO_2-B nanorod**	**AC**	~23	~2,800	0 – 2.8	1.0 M $LiPF_6$ in EC/DMC	[53]
**H-TiO_2-PPy-SCNT**	**AC**	~31	~4,000	1.0 – 3.0	-	[43]
**RuO_2/TiO_2 nanotube**	**AC**	~13	~1,200	0 – 1.4	1.0 M KOH	[54]
